# Characterization of fowl adenovirus isolates in Taiwan: genome, antigenicity, and pathogenicity

**DOI:** 10.1186/s13567-026-01793-z

**Published:** 2026-07-09

**Authors:** Lu Peng, Min Liu, Ming-Chu Cheng, Hui-Wen Chen

**Affiliations:** 1 Department of Veterinary Medicine, National Taiwan University, 1 Sec 4 Roosevelt Road, Taipei, 10617 Taiwan; 2 Animal Resource Center, National Taiwan University, Taipei, Taiwan; 3 Department of Veterinary Medicine, National Pingtung University of Science and Technology, Pingtung, Taiwan

**Keywords:** Fowl adenovirus, whole genome sequencing, antigenicity, pathogenicity, co-infection

## Abstract

Fowl adenoviruses (FAdVs) are important avian pathogens associated with inclusion body hepatitis and hydropericardium-hepatitis syndrome, contributing to significant economic losses in the poultry industry. Despite an increasing number of cases in Taiwan, the comprehensive characterization of circulating strains remains limited. This study investigated the molecular features, antigenic properties, and pathogenicity of FAdVs isolated in Taiwan between 2020 and 2025. Among suspected cases from broiler, layer, breeder, and colored broiler flocks, ten FAdV isolates were obtained. Whole-genome sequencing revealed six serotypes (FAdV-2, -3, -4, -8a, -8b, and -11) belonging to three species (C, D, and E), with one isolate exhibiting intertypic recombination. Cross-neutralization assays confirmed distinct serotypes with minimal antigenic overlap, highlighting the diversity of FAdVs circulating in Taiwan. A representative strain (NTU/C1120/2023) was selected for experimental infection in specific-pathogen-free chickens to evaluate pathogenicity. The isolate induced mild clinical signs and characteristic hepatic lesions in infected birds. Histopathology and immunohistochemistry further confirmed marked hepatic involvement. In addition, co-infection experiments with infectious bronchitis virus (IBV) were conducted to investigate potential interactions between the two viruses. Under co-infection conditions, FAdV viral loads in tissues were lower, whereas viral shedding tended to persist longer and antibody responses were reduced. In vitro assays using chicken embryo kidney cells further demonstrated reciprocal effects on viral replication. Collectively, this study provides new insights into the genetic and antigenic diversity of FAdVs in Taiwan and suggests that interactions with IBV may influence viral replication dynamics.

## Introduction

Fowl adenoviruses (FAdVs) are non-enveloped, icosahedral, double-stranded DNA viruses of the genus *Aviadenovirus* within the family *Adenoviridae* [1]. They are widely distributed in poultry and are implicated in several clinical syndromes, including inclusion body hepatitis (IBH) and hydropericardium hepatitis syndrome (HHS), which contribute to substantial economic losses in the poultry industry [2, 3]. Outbreaks in young chickens are often characterized by growth retardation, elevated mortality, and impaired production performance [3]. FAdV virions are composed of three major structural proteins: hexon, fiber, and penton [4]. Among these, the hexon serves as the primary antigenic determinant and a reliable molecular marker for phylogenetic genotyping [5]. Based on hexon gene sequence analysis, FAdVs are classified into five species (FAdV-A to FAdV-E) and twelve serotypes (FAdV-1 to FAdV-11, including 8a and 8b) [6]. Recently, the International Committee on Taxonomy of Viruses has adopted Latinized binomial names for these species, designating them as *Aviadenovirus ventriculi*, *Aviadenovirus quintum*, *Aviadenovirus hydropericardii*, *Aviadenovirus gallinae*, and *Aviadenovirus hepatitidis* [7].

In the past 10 years, FAdV infections have been increasingly reported worldwide, particularly across Asia, Europe, and the Americas, including cases in Poland [8], Iran [9], Spain [1, 10], Japan [11, 12], South Korea [13], China [14], the United States [15], and Brazil [16]. The simultaneous circulation of multiple serotypes within commercial poultry populations has complicated field diagnosis and control efforts [17]. Among these, serotypes within species D and E are most frequently associated with IBH, while serotype 4 of species C is typically linked to HHS [2, 18]. Furthermore, FAdVs are often detected alongside other immunosuppressive pathogens such as infectious bursal disease virus (IBDV) [19, 20] and chicken anemia virus (CAV) [17, 21, 22], as well as infectious bronchitis virus (IBV) [23], which is highly prevalent in poultry populations. These co-infections can exacerbate clinical severity, impair immune competence, and alter viral replication dynamics, posing additional challenges to vaccination and biosecurity programs. Despite these observations, the factors influencing FAdV pathogenesis and virus–virus interactions remain largely undefined, particularly with respect to interactions between FAdV and IBV and their impact on viral replication dynamics.

Although FAdV-associated diseases have been recognized in Taiwan for several decades, comprehensive investigations into the molecular characteristics, antigenic relationships, and pathogenicity of local strains are scarce. Recent diagnostic surveillance has revealed the re-emergence and concurrent circulation of multiple serotypes among broiler, layer, and breeder flocks, suggesting ongoing viral evolution and endemic persistence. The coexistence of genetically distinct lineages raises concerns regarding antigenic variation, intertypic recombination, and the limited cross-protection provided by existing monovalent vaccines [24]. Given the increasing complexity of FAdV epidemiology, region-specific molecular and serological characterization is essential for guiding effective prevention and control strategies.

The present study aimed to elucidate the genetic and antigenic diversity of FAdV isolates circulating in Taiwan from 2020 to 2025 and to assess their pathogenicity in specific-pathogen-free (SPF) chickens. Representative isolates were subjected to full-genome sequencing, phylogenetic and recombination analyses, cross-neutralization assays, and experimental infection models. In addition, a representative strain was used to investigate the effects of co-infection with infectious bronchitis virus (IBV), which is highly prevalent in Taiwan and frequently co-detected with FAdV [23], on viral replication dynamics. Collectively, these findings provide new insights into the molecular epidemiology, antigenic variation, and pathogenesis of FAdVs in Taiwan and establish a foundation for the development of improved diagnostic tools and targeted vaccination strategies.

## Materials and methods

### Source of clinical cases and molecular detection of the causative virus

From January 2020 to June 2025, suspected cases of FAdV infection were collected from poultry farms across Taiwan, including breeders, broilers, and layers. For each case, approximately three chickens were necropsied, and liver tissues with prominent gross lesions were selected. One portion was fixed in 10% neutral-buffered formalin for histopathological examination (H&E staining), while the other was homogenized in Waymouth MB 752/1 medium (Merck, Germany), clarified at 3000 × *g* for 10 min, and used for nucleic acid extraction and virus isolation. Conventional PCR targeting the hexon gene was performed using primers FAdV-HexonA (5′-CAARTTCAGRCAGACGGT-3′) and FAdV-HexonB (5′-TAGTGATGMCGSGACATCAT-3′) as described by the previous study [25]. Amplicons (~897 bp) were analyzed by 2% agarose gel electrophoresis, and positive samples were sequenced (TRI Biotech, New Taipei, Taiwan).

### Virus identification by ICC staining in LMH cells

To further confirm virus identity, immunocytochemical (ICC) staining was performed on infected Leghorn male hepatoma (LMH) cells as previously described with modifications [26]. Cells were fixed with 80% acetone at −20 °C, blocked with 5% goat serum, and incubated with FAdV-positive chicken serum (1:100 dilution) as the primary antibody, followed by HRP-conjugated rabbit anti-chicken IgG (1:400; Jackson ImmunoResearch, USA). Color development was achieved with DAB substrate (DAKO, USA), and hematoxylin was used for nuclear counterstaining. Cells exhibiting brown intracellular staining were considered FAdV-positive.

### Virus isolation, propagation and titration

FAdV-positive samples were propagated using the LMH cell line (ATCC CRL-2177^™^). Liver homogenates stored at −80 °C were subjected to three freeze–thaw cycles, clarified by centrifugation at 3000 × *g* for 10 min, and filtered through sterile 0.45 µm syringe filters (Pall Life Sciences, USA). The filtrates were inoculated onto confluent LMH monolayers grown in gelatin-coated flasks and adsorbed for 2 h at 37 °C in 5% CO₂. Cultures were maintained in Waymouth MB 752/1 medium supplemented with 2% fetal bovine serum (Gibco, USA) and 1% PSA (penicillin–streptomycin–amphotericin B; Sigma-Aldrich, USA), and were examined daily for cytopathic effects (CPE). Upon observation of CPE, culture supernatants were collected and passaged once more in LMH cells. After two passages, isolates confirmed as FAdV-positive by PCR were considered successfully recovered. For virus propagation, LMH cells in T75 flasks were infected and incubated at 37 °C with 5% CO₂ until extensive CPE was observed. Both cells and supernatants were harvested, subjected to three freeze–thaw cycles, clarified by centrifugation, aliquoted, and stored at −80 °C as viral stocks. Virus titers were determined in LMH cells seeded in 96-well plates (1 × 10^4^ cells/well). Viral suspensions were serially diluted tenfold (10⁻^1^–10⁻⁹) with eight replicates per dilution and inoculated (100 µL/well). After 2 h of adsorption, the inoculum was replaced with maintenance medium, and plates were incubated at 37 °C with 5% CO₂. CPE were evaluated at 5 dpi, and TCID₅₀ values were calculated using the Reed and Muench method [27].

### Whole-genome sequencing and phylogenetic analysis

Viral genomes were sequenced using an Oxford Nanopore MinION platform with FLO-MIN106D (R9.4.1) flow cells. Library preparation and sequencing were performed according to the manufacturer’s instructions. Basecalling was conducted with Guppy, and de novo genome assembly was carried out using Flye, followed by polishing with Medaka. Complete genome sequences were aligned with representative FAdV references retrieved from GenBank using ClustalW. Phylogenetic trees were inferred in MEGA 11 using the neighbor-joining method with 1000 bootstrap replicates and visualized in iTOL [28]. Recombination analysis was performed with SimPlot v3.5.1 using similarity plot and bootscan methods (window size 500 bp, step size 20 bp, Kimura 2-parameter model, transition/transversion ratio = 2.0) [29].

### Transmission electron microscopy

Virus particles propagated in LMH cells were clarified at 3000 × *g* for 20 min and pelleted by ultracentrifugation at 40 000 × *g* for 3 h at 4 °C. Pellets were resuspended in TEN buffer (50 mM Tris–HCl, 1 mM EDTA, 100 mM NaCl; pH 7.4). Ten µL suspensions were applied onto carbon-coated copper grids, stained with 1% phosphotungstic acid (pH 7.0), air-dried, and examined with a transmission electron microscope (JEM-1400, JEOL, Japan) [26].

### Hyperimmune serum preparation and cross-neutralization assay

Hyperimmune sera were generated in BALB/c mice by intramuscular immunization with UV-inactivated whole FAdV derived from representative isolates identified in this study for each serotype (10^8^ TCID₅₀ per dose) emulsified with Freund’s adjuvant (complete for the prime, incomplete for two boosters at 3-week intervals). Complete inactivation of FAdV was confirmed by inoculation onto LMH cells, where no cytopathic effects were observed after incubation. Two weeks after the final boost, sera were collected, heat-inactivated (56 °C for 30 min), aliquoted, and stored at −20 °C. Cross-neutralization assays were performed by mixing serial four-fold dilutions of sera with 100 TCID₅₀ of virus for 1 h at 37 °C, followed by inoculation onto confluent LMH monolayers in 96-well plates. Cultures were monitored for CPE for 5–7 days. Neutralizing titers were defined as the highest dilution protecting ≥ 50% of wells. Antigenic relationships were determined by r-values [30]. Values lower than 10% were considered as distinct serotypes [31]. All animal procedures were approved by the Institutional Animal Care and Use Committee (IACUC) of National Taiwan University (Approval No. NTU-111-EL-00093).

### Pathogenicity examination

One-day-old SPF white Leghorn chickens were randomly allocated into three groups: control (*n* = 8), FAdV-infected (*n* = 12), and co-infected with FAdV and IBV (*n* = 18). Birds were housed in separate isolators with feed and water ad libitum. The FAdV group received 10⁷ TCID₅₀ of strain NTU/C1120/2023 (FAdV-8a) orally, while the co-infected group was simultaneously inoculated with the same FAdV strain (10⁷ TCID₅₀, oral) and 10⁶ EID₅₀ of IBV strain S78 (intranasal), which was isolated in a previous study [31]. Clinical signs, body weight, and mortality were recorded daily and scored on a 0–4 scale (0 = normal; 1 = mild depression; 2 = severe depression; 3 = paralysis or prostration; 4 = death) to evaluate the pathogenicity of the FAdV isolate in SPF chickens. Oropharyngeal and cloacal swabs, as well as blood samples, were collected at 3, 5, 7, 11, 14, 21, and 28 dpi for viral shedding and antibody analysis. At 7, 14, 21, and 28 dpi, three birds from each infected group were necropsied, and tissues (bursa, thymus, heart, liver, spleen, and kidney) were collected. Histopathological examination (H&E and IHC) was performed in the FAdV-infected group to characterize lesions and viral antigen distribution associated with FAdV infection. For molecular analysis, viral DNA loads in tissues and swabs were quantified by quantitative PCR to evaluate viral replication dynamics under single infection and co-infection conditions. All animal procedures were approved by the IACUC of National Taiwan University (Approval No. NTU-114-EL-00018).

### Quantitative PCR assays

Nucleic acids were extracted from tissue samples and swabs using the taco^™^ DNA/RNA Extraction Kit (GeneReach, Taiwan) according to the manufacturer’s instructions. The extracted DNA was used for quantification of FAdV by SYBR Green-based qPCR targeting the 52 K gene on a CFX Connect Real-Time PCR Detection System (Bio-Rad, USA). Primers adapted from a previous study [32] amplified a 191-bp fragment (forward: 5′-ATGGCKCAGATGGCYAAGG-3′; reverse: 5′-GGAGGYCKGTTCTCGAGCG-3′). Reactions were prepared with QuantiNova SYBR Green PCR Master Mix (QIAGEN), and cycling was 95 °C for 2 min, followed by 44 cycles of 95 °C for 5 s and 60 °C for 10 s, with melt curve analysis to confirm specificity.

For IBV detection, extracted RNA was analyzed by a TaqMan-based RT-qPCR targeting the 3′ untranslated region (3′UTR) on the same platform. Primers and probe were developed in our laboratory and are described as follows: forward 5′-TCGCCAGGGAAATGTCTAATC-3′, reverse 5′-GGCACTGGCATCTTTCTAACTA-3′, and FAM-labeled probe 5′-AGGGTCTACCTTTCGTTTCCAGGC-3′. Reactions were prepared with PrimeTime Gene Expression Master Mix (IDT) and run at 95 °C for 2 min, followed by 45 cycles of 95 °C for 5 s and 60 °C for 30 s.

### Enzyme-linked immunosorbent assay (ELISA)

Collected serum was diluted 1:100 and tested for antibodies against fowl adenovirus using a commercial Fowl Adenovirus Group 1 Antibody Test Kit (BioChek, Netherlands), following the manufacturer’s instructions. Samples with a sample-to-positive (S/P) ratio greater than 0.5 were considered seropositive.

### Immunohistochemistry (IHC)

Paraffin-embedded tissue sections (5 µm) were deparaffinized, rehydrated, and subjected to antigen retrieval in 10 mM citrate buffer (pH 6.0) at 95 °C for 20 min. Endogenous peroxidase activity was blocked with 3% hydrogen peroxide, and slides were incubated with 1% BSA in PBST. Sections were probed with mouse hyperimmune serum against FAdV (1:200) for 2 h at room temperature, followed by HRP-conjugated goat anti-mouse IgG (Jackson ImmunoResearch). Signal was developed with DAB and counterstained with hematoxylin.

### In vitro co-infection experiment in CEK cells

Primary chicken embryo kidney (CEK) cells were prepared from 19-day-old SPF embryos and maintained in M199 medium (Gibco, USA) supplemented with 10% FBS and 1% PSA. Cells were seeded in 96-well plates and infected at 90–100% confluence under six conditions: FAdV-only, IBV-only, FAdV → IBV, IBV → FAdV, simultaneous co-infection, and uninfected control. FAdV NTU/C1120/2023, a field isolate belonging to serotype 8a identified in this study, was used at a multiplicity of infection (MOI) of 1, while IBV S78, a previously characterized virulent strain isolated in Taiwan [31], was used at 10⁶ EID₅₀. For sequential infections, the second virus was added 4 h after the first. After adsorption for 1 h in M199 medium containing 2% FBS, the inoculum was removed and cells were maintained in M199 supplemented with 10% FBS. Cytopathic effects (CPE) were monitored at 24, 48, and 72 h post-infection (hpi), and culture supernatants were collected for nucleic acid extraction. FAdV replication was quantified by qPCR targeting the 52 K gene, whereas IBV replication was determined by RT-qPCR targeting the 3′ untranslated region (3′UTR). All experimental conditions were performed in duplicate.

### Statistical analysis

All statistical analyses were conducted using GraphPad Prism v10 (GraphPad Software, San Diego, CA, USA). Data are presented as mean ± SEM. Comparisons between two groups were performed using unpaired Student’s *t*-test or Mann–Whitney *U* test, depending on data distribution. For comparisons involving more than two groups, one-way or two-way ANOVA was applied. A *p* < 0.05 was considered statistically significant.

## Results

### Clinical cases and virus detection

**Figure 1:**
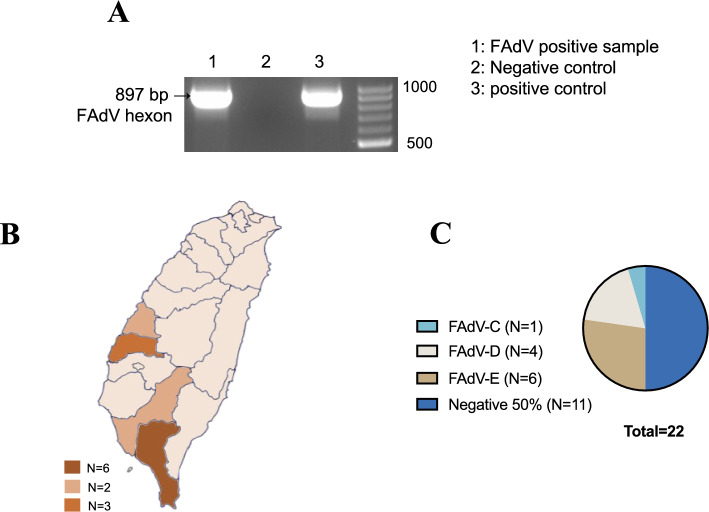
**Detection and distribution of FAdV in clinical samples from Taiwan. A** Agarose gel electrophoresis showing the PCR amplification of the FAdV hexon gene (897 bp). Lane 1: FAdV-positive sample; Lane 2: Negative control; Lane 3: Positive control. **B** Geographic distribution of FAdV-positive cases in Taiwan. **C** Pie chart summarizing the FAdV species distribution among 22 clinical samples.

A total of 22 suspected cases of FAdV infection were submitted, including 15 from the National Taiwan University Veterinary Medicine Diagnostic Center (Yunlin Branch) and 7 from the National Pingtung University of Science and Technology. Gross lesions consisted of hepatomegaly and pallor of the liver. Microscopically, all cases showed multifocal to diffuse hepatocellular necrosis, and basophilic intranuclear inclusion bodies were observed in four cases. PCR screening identified 11 positive cases (50%) (Figure 1A), including broilers (*n* = 6), colored broilers (*n* = 3), and layers (*n* = 2). Positive cases were distributed across central and southern Taiwan (Figure 1B). Sanger sequencing of PCR amplicons classified the isolates as FAdV-C (*n* = 1), FAdV-D (*n* = 4), and FAdV-E (*n* = 6) (Figure 1C). Reported mortality in affected flocks ranged from 0.3% to 10%. Clinical onset occurred at 2–4 weeks of age in broilers and 16–18 weeks in layers. Detailed case information is summarized in Table 1.

**Table 1:** **Summary of field cases of FAdV infection investigated in this study**

Date (yyyy/mm)	Age	Mortality (%)	Breed	Location	Clinical signs	Gross lesions	Microscopic lesions	FAdV isolate (Genotype)
2020/02	70d	0.08	Colored broiler	Pingtung	Mortality rate increases	Liver enlarged, diffuse white spots on the surface	Hepatocyte necrosis, fatty change	NTU/C1921/2024 (D)
2020/03	65d	0.06	Colored broiler	Pingtung	Depression	Liver enlarged, greenish discoloration	Hepatocyte necrosis, fatty change	NTU/C1922/2024 (D)
2020/03	18w	0.13	Layer	Pingtung	Mortality rate increases	Liver and pancreas enlarged	Hepatocyte necrosis, fatty change	NTU/C1923/2024 (E)
2021/07	23d	3	Colored broiler	Pingtung	Mortality rate increases	Liver with yellow discoloration	Hepatocyte necrosis, fatty change, Intranuclear inclusion body	NTU/C1634/2024 (E)
2022/12	23d	10	Broiler	Yunlin	Mortality rate increases, loss of appetite	Liver mottled and enlarged with round edge	Intranuclear inclusion body	NTU/C924/2022 (D)
2023/03	19d	0.60	Broiler	Pingtung	Mortality rate increases	Liver enlarged, pale, with multiple white spots on the surface	Hepatocyte necrosis, fatty change, Intranuclear inclusion body	NTU/C1635/2024 (E)
2023/03	13d	2.10	Broiler	Taichung	Mortality rate increases	Pale liver with white foci	Hepatocyte necrosis	NTU/C1005/2023 (E)
2023/06	20d	0.30	Broiler	Yunlin	Mortality rate increases	Liver enlarged	Hepatocyte necrosis	NTU/C1120/2023 (E)
2023/11	8d	1.06	Broiler	Pingtung	Mortality rate increases	Liver enlarged	Hepatocyte necrosis, Intranuclear inclusion body	NTU/C1636/2024 (D)
2024/05	16w	0.04	Layer	Yunlin	Mortality rate increases	Liver enlarged with multiple white spots on the surface	Hepatocyte necrosis	NTU/C1648/2024 (C)

### Virus characterization in LMH cells

**Figure 2:**
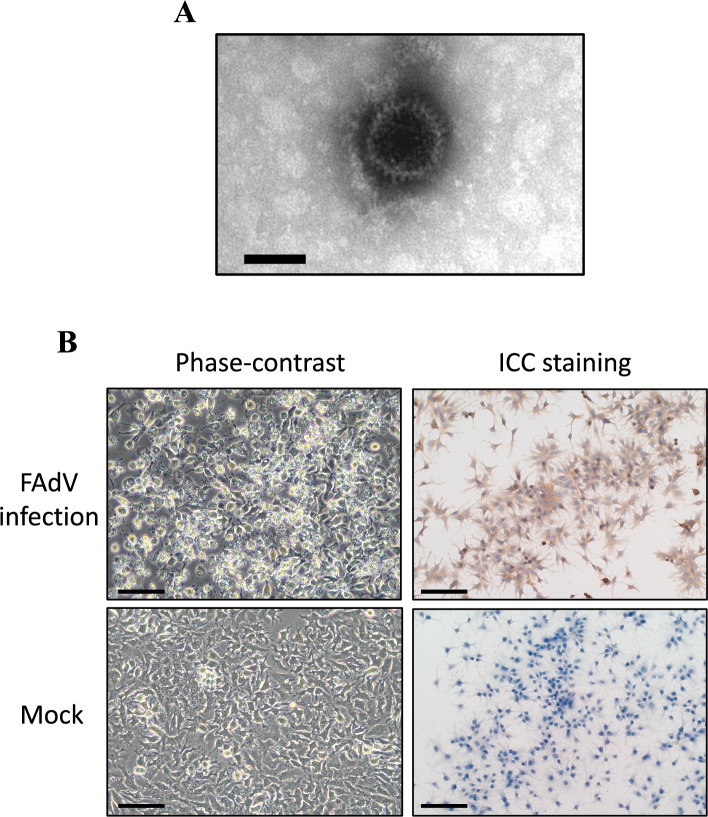
**Characterization of FAdV infection in LMH cells. A** TEM of viral preparations from LMH cells infected with isolate NTU/C1005/2023. Scale bar = 100 nm. **B** ICC detection of FAdV antigen expression in LMH cells. Images were taken under phase-contrast microscopy at 200 × magnification. Scale bar = 100 µm.

Among the eleven PCR-positive cases of FAdV infection, ten virus isolates were successfully obtained using LMH cell culture (Table 1), while one case did not yield a viable isolate. Transmission electron microscopy (TEM) of viral preparations from LMH cells infected with isolate NTU/C1005/2023 showed non-enveloped, icosahedral virions measuring 70–90 nm in diameter (Figure 2A). Infected LMH cells exhibited progressive cytopathic effects, including cell rounding and detachment of the monolayer (Figure 2B). Immunocytochemistry (ICC) using FAdV-positive chicken serum revealed strong brown cytoplasmic staining, predominantly in the perinuclear region, whereas mock-infected controls showed no signal (Figure 2B).

### Whole genome sequencing and annotation

Ten FAdV isolates underwent whole genome sequencing using the Oxford Nanopore Technologies (ONT) platform. High-quality genomes were assembled for all isolates, with lengths ranging from 43 320 to 45 640 bp and GC contents between 53 and 58%. Genome annotation identified 31–41 predicted ORFs per isolate, including structural genes (hexon, penton base, fiber), replication-related genes (DNA polymerase, IVa2), and assembly-associated genes (100K, 52 K, pVI). No evidence of mixed infection was detected based on Sanger sequencing and whole genome read-mapping analysis. Complete genome sequences were deposited in GenBank (accession numbers PV737789–PV737798).

### Phylogenetic analysis

**Figure 3:**
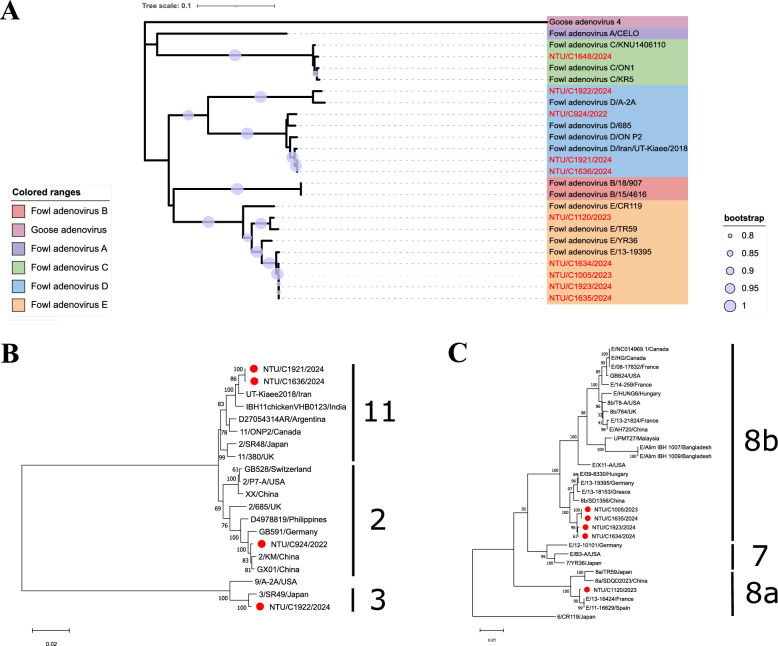
**Phylogenetic analysis of FAdV isolates based on whole-genome and hexon gene sequences. A** phylogenetic tree constructed from complete genome sequences of representative FAdV and goose adenovirus strains. **B** phylogenetic tree of FAdV-D based on full-length hexon gene sequences (2844 bp). **C** phylogenetic tree of FAdV-E based on full-length hexon gene sequences (2844 bp). Isolates obtained in this study are shown in red, and the phylogenetic trees were constructed using the neighbor-joining method with 1000 bootstrap replicates.

Phylogenetic relationships were reconstructed using representative adenovirus sequences obtained from NCBI. Based on whole-genome nucleotide identity, the isolates were classified into three species within the genus *Aviadenovirus*: FAdV-C (*n* = 1), FAdV-D (*n* = 4), and FAdV-E (*n* = 5) (Figure 3A). Further phylogenetic analysis of the full-length hexon gene revealed that the FAdV-D isolates clustered into serotypes 2, 3, and 11 (Figure 3B), whereas the FAdV-E isolates were assigned to serotypes 8a and 8b (Figure 3C). The isolate NTU/C1648/2024 belonged to species FAdV-C and clustered with FAdV-4 reference strains such as GX2019-014 (China), showing more than 99% nucleotide identity across the genome and major structural genes. Four isolates, including NTU/C924/2022, NTU/C1636/2024, NTU/C1921/2024, and NTU/C1922/2024 were grouped within FAdV-D, clustering with serotype 2 and 3 reference strains such as GX01, SR48, P7-A, and SR49, and exhibiting up to 99.6% identity in the hexon gene. The remaining five isolates, NTU/C1005/2023, NTU/C1634/2023, NTU/C1635/2024, NTU/C1923/2024, and NTU/C1120/2023, were classified as FAdV-E. Among them, NTU/C1005/2023, NTU/C1634/2023, NTU/C1635/2024, and NTU/C1923/2024 clustered with serotype 8b reference strains such as SDQD2023 and HeB20, sharing more than 98.9% identity in the hexon gene sequences.

### Recombination analysis

**Figure 4:**
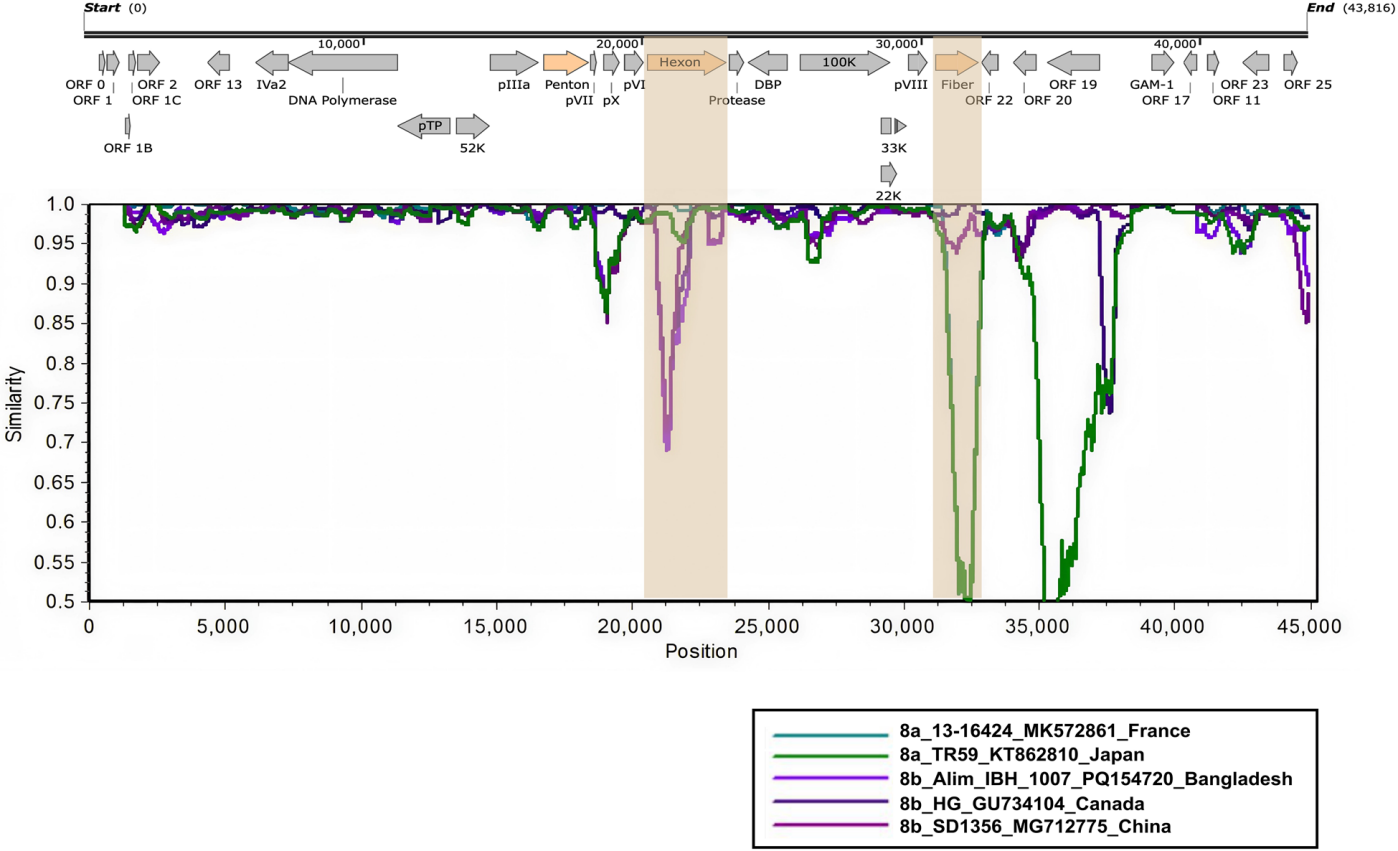
**Recombination analysis and whole-genome similarity of FAdV isolate NTU/C1120/2023.** SimPlot analysis was performed using the NTU/C1120/2023 genome as the query sequence to assess nucleotide similarity against representative FAdV-E reference strains, including FAdV-8a TR59 (Japan), FAdV-8b SD1356 (China), FAdV-8b Alim-IBH-1007 (Bangladesh), FAdV-8b HG (Canada), and FAdV-E 13–16424 (Germany). The upper panel depicts the genomic organization of NTU/C1120/2023, with annotated ORFs and key structural genes (hexon, fiber, and penton) highlighted. The lower panel shows the nucleotide similarity plot across the entire genome.

To further examine genetic variation among the isolates, recombination analysis was performed based on complete genome sequences using SimPlot v3.5.1. Among the ten isolates, NTU/C1120/2023 exhibited a distinct recombination pattern. As shown in Figure 4, similarity plot analysis revealed that the hexon gene region (nucleotide positions 20 000–23 000) shared the highest sequence similarity with the FAdV-8a reference strain TR59_8a (Japan), whereas the fiber gene region (approximately positions 31 000–32 000) showed greater similarity to the FAdV-8b strain SD1356_8b (China). The crossover points were located within the two major capsid protein genes, indicating the presence of an 8a-like hexon and an 8b-like fiber within the same genome. This genomic structure suggests that the isolate contains segments derived from both serotypes, forming a mosaic genome architecture characteristic of recombination between FAdV-8a and FAdV-8b lineages.

### Cross-neutralization test

To assess antigenic relationships among circulating FAdV isolates, cross-neutralization assays were performed using antisera raised against six representative serotypes. The neutralization capacity was evaluated on LMH cells, and r-values were calculated by setting homologous reactions as 100. The results (Table 2) demonstrated that each antiserum reacted strongly with its homologous strain but showed negligible activity against heterologous serotypes. The low r-values observed across most pairwise comparisons, with the highest at 2.37% between FAdV-2 and FAdV-11, suggest that these isolates represent antigenically distinct serotypes.

**Table 2:** **Cross-neutralization test among representative FAdV isolates from different serotypes**

FAdV isolate	C1923 (8b)	C1636 (11)	C1648 (4)	C924 (2)	C1922 (3)	C1120 (8a)
C1923 (8b)	**100**					
C1636 (11)	0.01	**100**				
C1648 (4)	0.28	0.02	**100**			
C924 (2)	0.02	2.37	0.02	**100**		
C1922 (3)	0.02	0.18	0.07	0.01	**100**	
C1120 (8a)	2.2	0.15	0.1	0.02	0.03	**100**

C1923: NTU/C1923/2024, C1636: NTU/C1636/2024, C1648: NTU/C1648/2024, C924: NTU/C924/2022, C1922: NTU/C1922/2024, C1120: NTU/C1120/2023. Bold numbers indicate homologous r-values.

### Pathogenicity in SPF chickens

**Figure 5:**
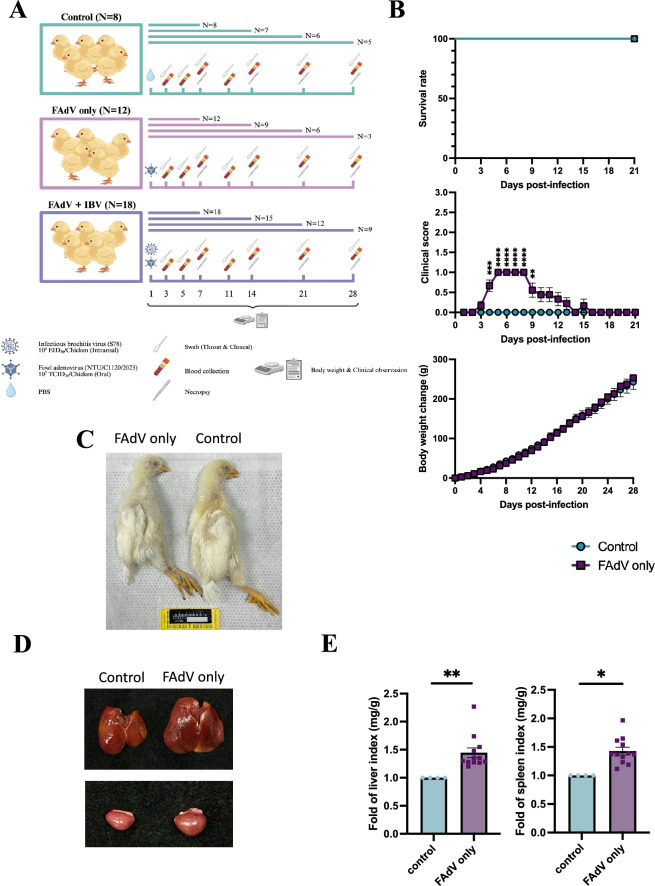
**Pathogenicity of FAdV infection in SPF chickens. A** Schematic overview of the experimental design. **B** Survival rate, clinical scores, and body weight changes in control and FAdV-only groups during infection. **C** Representative appearance of chickens from the FAdV-only and control groups at necropsy. **D** Gross lesions of the liver and spleen in control and FAdV-only groups. **E** Fold changes in liver and spleen indices (organ weight/body weight, mg/g) relative to controls. Each dot represents an individual bird. Data are presented as mean ± SEM. Statistical comparisons were performed using an unpaired Student’s *t*-test (*p < 0.05, **p < 0.01, ***p < 0.001, ****p < 0.0001).

**Figure 6:**
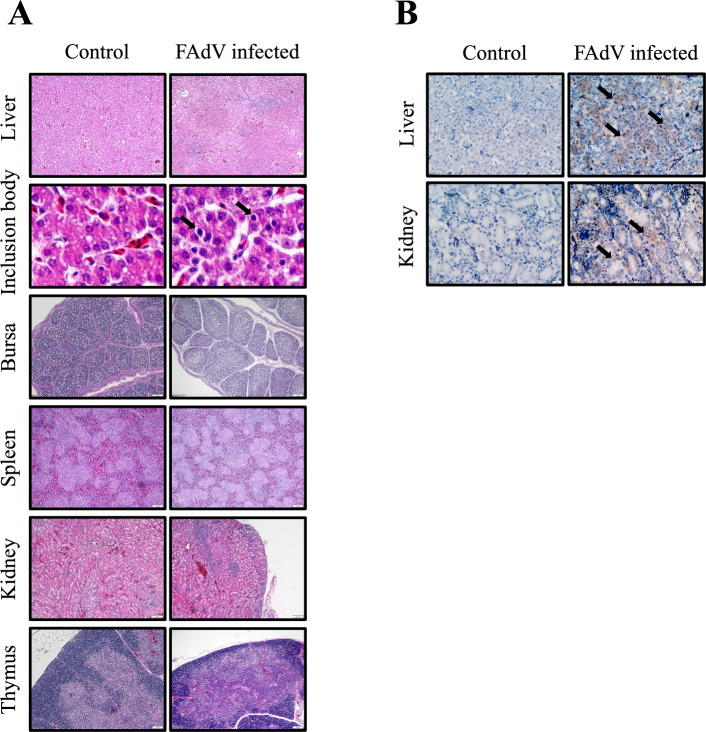
**Histopathological and immunohistochemical findings in chickens infected with FAdV. A** Representative hematoxylin and eosin (H&E)-stained sections of liver, bursa of Fabricius, spleen, kidney, and thymus from control and FAdV-infected chickens. Hepatocellular necrosis and basophilic intranuclear inclusion bodies were observed in the liver of infected birds (arrows). **B** Immunohistochemical detection of FAdV antigens in liver and kidney. Positive staining is indicated by brown coloration (arrows).

**Figure 7:**
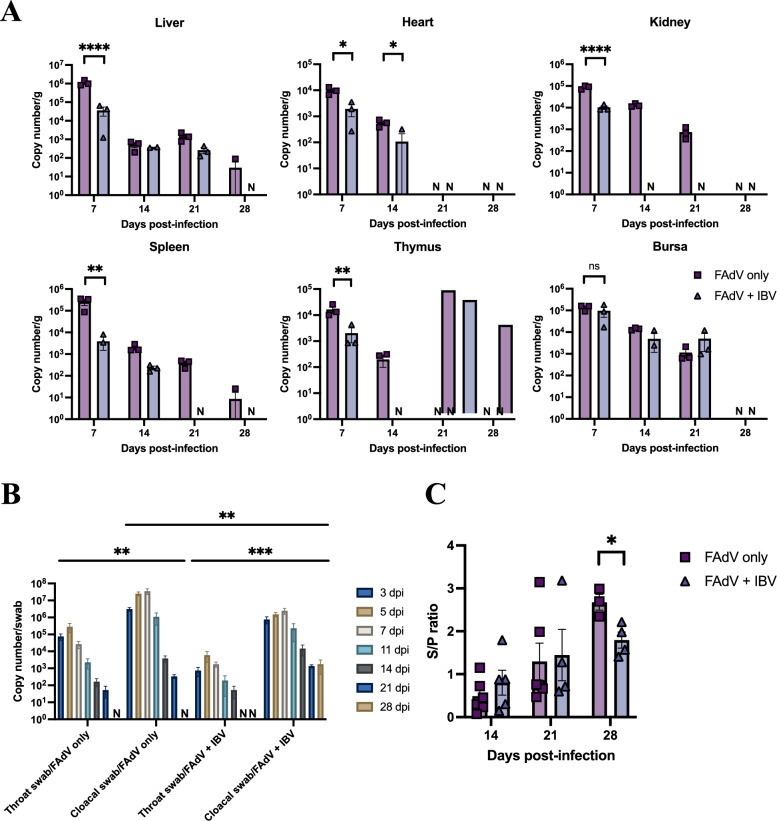
**Tissue viral load, viral shedding, and antibody response in chickens infected with FAdV or co-infected with IBV. A** Tissue viral load in liver, heart, kidney, spleen, thymus, and bursa of Fabricius at 7, 14, 21, and 28 dpi, quantified by qPCR. Each dot represents an individual bird; “N” indicates samples with undetectable virus. **B** Viral shedding in throat and cloacal swabs collected at 3–28 dpi. Viral genome copies were determined by qPCR. **C** Serum antibody responses against FAdV measured by ELISA, expressed as sample-to-positive (S/P) ratios. Data are presented as mean ± SEM. Statistical comparisons were performed using unpaired Student’s *t*-test and two-way ANOVA (*p < 0.05, **p < 0.01, ***p < 0.001, ****p < 0.0001).

The experimental design is shown in Figure 5A. Chickens infected with FAdV developed mild clinical signs approximately 1 week post-infection, including slight depression and ruffled feathers, which gradually resolved during the following week without apparent complications (Figure 5B). No mortality was observed in the FAdV-infected group. Body weight gain did not differ significantly between the FAdV-infected and control groups throughout the experimental period. However, greater variability in body weight gain was observed in the FAdV-infected group at later time points (Figure 5B). At necropsy, no obvious growth retardation was observed, and the general appearance of FAdV-infected chickens was comparable to that of controls (Figure 5C). Mild hepatomegaly and splenomegaly were observed in the FAdV-infected group, accompanied by significantly increased liver and spleen indices compared with controls (*p* < 0.05). Multifocal pale white lesions were also present along the hepatic margins, whereas these gross changes were not observed in the control group (Figure 5D, E). Histopathological examination revealed hepatic necrosis and lymphoid depletion in the bursa, spleen, and thymus in FAdV-infected chickens (Figure 6A). No obvious lesions were observed in the kidney. Immunohistochemical staining confirmed FAdV antigen localization mainly within hepatocytes and renal tubular epithelial cells at 7 dpi (Figure 6B). To further evaluate viral replication dynamics, quantitative PCR analysis was performed. FAdV DNA was detectable in all examined tissues, peaking at 7 dpi and subsequently declining (Figure 7A). The liver showed the highest viral loads, followed by the spleen and thymus, while viral DNA persisted in the bursa up to 21 dpi. In addition, viral shedding and antibody responses were compared between infection groups. Cloacal viral shedding exceeded oropharyngeal shedding in magnitude and duration, persisting until 28 dpi in the co-infected group (Figure 7B). ELISA detected anti-FAdV antibodies from 14 dpi onward, with significantly lower titers observed in the co-infected group at 28 dpi (*p* < 0.05) (Figure 7C).

### In vitro co-infection of FAdV and IBV in CEK cells

**Figure 8:**
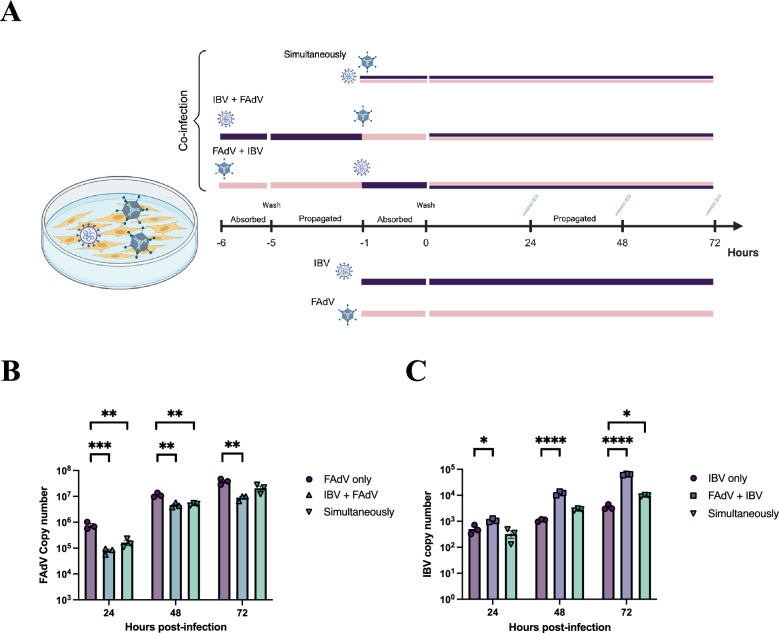
**Viral replication kinetics of FAdV and IBV in CEK cells under different co-infection conditions. A** Schematic overview of the co-infection experimental design in CEK cells. **B** FAdV genome copy numbers quantified by qPCR at hpi 24, 48, and 72 under different infection conditions. **C** IBV genome copy numbers were determined by qPCR at the same time points. Data are shown as mean ± SEM from three replicates. Statistical analysis was performed using two-way ANOVA followed by Tukey’s multiple comparisons test (*p < 0.05, **p < 0.01, ***p < 0.001, ****p < 0.0001).

To investigate the impact of co-infection on viral replication dynamics, CEK cells were infected under different conditions, and viral replication kinetics were analyzed (Figure 8A). FAdV replication peaked at 72 h in the FAdV-only group. Lower FAdV replication levels were observed under both sequential (IBV before FAdV) and simultaneous co-infection conditions (*p* < 0.05) (Figure 8B). In contrast, IBV replication levels were significantly increased in the sequential (FAdV before IBV) co-infection group compared with the IBV-only group (*p* < 0.05) (Figure 8C), suggesting an interaction between the two viruses at the level of replication dynamics. No viral genomes were detected in uninfected controls.

## Discussion

This study provides a comprehensive molecular and pathological characterization of FAdVs currently circulating in Taiwan. Between 2020 and 2025, eleven FAdVs representing three genotypes (FAdV-C, -D, and -E) and six serotypes (FAdV-2, −3, −4, −8a, −8b, and −11) were identified from poultry farms exhibiting clinical signs consistent with inclusion body hepatitis. These findings reveal a high level of genetic and antigenic heterogeneity within local FAdV populations and indicate a shift in the dominant genotypes compared with those reported two decades ago. According to diagnostic records from National Taiwan University in the early 2000 s, FAdV genotypes A and C were previously predominant in local poultry flocks [33]. In contrast, the present study identified genotypes D and E as the dominant types, suggesting a notable shift in the population structure of circulating FAdVs. Similar trends of serotype replacement have been reported in other Asian regions, such as Korea [34], where FAdV-4 predominated before 2015 but was later replaced by FAdV-8b and −11, and Spain [1], where long-term surveillance revealed FAdV-8b and −11 as the principal types associated with IBH outbreaks. Together, these results suggest that the FAdV population in Taiwan is undergoing active evolution, warranting continued surveillance and genomic monitoring to track emerging strains.

An intertypic recombinant isolate, NTU/C1120/2023, was identified in this study, with the hexon and fiber genes derived from distinct parental genotypes. Recombination within structural genes, particularly hexon and fiber, has been increasingly recognized as a major driver of FAdV diversification and antigenic evolution [6]. Similar mosaic structures have been reported in China, including the AH720 and GDLZ strains, which exhibit reciprocal exchanges between FAdV-8a and −8b lineages [35, 36]. Such genetic rearrangements may modify epitope composition and facilitate immune escape, posing challenges to existing vaccines. Taken together, cross-neutralization assays in our study revealed minimal serological cross-reactivity among isolates, confirming their classification into distinct antigenic serotypes and underscoring the need for polyvalent or region-specific vaccine formulations.

Among the eleven FAdVs identified, nine originated from broiler flocks, suggesting that broilers are the primary hosts affected under field conditions in Taiwan. This observation is consistent with epidemiological data from other regions, where broilers account for the majority of clinical FAdV cases [17]. High stocking density, rapid growth, and immature immune systems may contribute to their increased susceptibility. Experimental studies have also demonstrated greater pathogenic responses in broilers compared with layers. When challenged with FAdV-D and -E isolates, broilers exhibited near-complete mortality, whereas layers showed only mild lesions and partial survival [37]. In our experimental infection model using the recombinant isolate NTU/C1120/2023, no mortality occurred, and clinical signs were mild, consistent with reports of low virulence in FAdV-8 lineage strains such as AH720 (no mortality during a 21-day observation period) and GDLZ (approximately 5% mortality) [35, 38]. Nevertheless, infected birds exhibited histological lesions in the liver, spleen, kidney, and bursa of Fabricius, accompanied by high viral loads in visceral and lymphoid tissues, indicating efficient replication despite limited clinical manifestation. Furthermore, comparative studies on FAdV serotypes [17] indicate that FAdV-4 is the most pathogenic serotype, followed by FAdV-11, while FAdV-8b exhibits the lowest virulence. Although NTU/C1120/2023 exhibited low virulence in single infections, co-infection experiments were conducted to explore potential interactions with other viral pathogens. In the present study, co-infection with infectious bronchitis virus (IBV) was associated with altered FAdV replication dynamics, as FAdV viral loads in tissues were lower in co-infected birds than in those infected with FAdV alone. Previous studies have reported that co-infections with viruses such as infectious bursal disease virus (IBDV), chicken anemia virus (CAV), and Newcastle disease virus (NDV) can influence FAdV infection outcomes [20, 22, 35]. A similar interaction has also been reported in dual infections involving different FAdV serotypes [36]. In vitro assays using chicken embryo kidney cells further supported these findings. Under co-infection conditions, lower FAdV replication levels were observed in the presence of IBV, consistent with the trend observed in the in vivo experiments. In contrast, IBV replication increased in the presence of FAdV in vitro. Together, these observations suggest that interactions between the two viruses may influence their replication dynamics and modulate host responses and replication efficiency. However, the absence of an in vivo IBV-only control group limits the ability to distinguish the contribution of IBV infection to the observed clinical signs and pathological changes; therefore, the impact of co-infection on disease severity should be interpreted with caution.

In conclusion, this study reveals the extensive genetic and antigenic diversity of FAdV strains currently circulating in Taiwan and highlights their distribution and pathogenic characteristics across different poultry production systems. Co-infection with other viral pathogens, particularly infectious bronchitis virus (IBV), was associated with altered viral replication dynamics. These findings emphasize several priorities for future research. Sustained nationwide molecular surveillance and genotyping are essential for tracking viral evolution, monitoring recombination events, and identifying newly emerging strains with potential epidemiological significance. Furthermore, for serotypes showing limited or no cross-neutralization, the development of multivalent or region-specific vaccines is necessary to improve protective efficacy. In addition, interactions between FAdV and other co-circulating pathogens should be considered when designing vaccination and control strategies, as co-infections may influence viral replication and host responses. Collectively, this study provides an updated understanding of FAdV epidemiology in Taiwan and supports the development of targeted prevention strategies to mitigate its impact on poultry health and production.

## Data Availability

All data generated or analyzed during this study are included in this published article.

## References

[1] Bertran K, Blanco A, Antilles N, Nofrarías M, Valle RM, Cobos À, Ramis A, Biarnés M, Majó N (2021) A 10-year retrospective study of inclusion body hepatitis in meat-type chickens in Spain (2011-2021). Viruses 13:2170 34834976 10.3390/v13112170 PMC8617850

[2] El-Shall NA, El-Hamid HSA, Elkady MF, Ellakany HF, Elbestawy AR, Gado AR, Geneedy AM, Hasan ME, Jaremko M, Selim S, El-Tarabily KA, El-Hack MEA (2022) Epidemiology, pathology, prevention, and control strategies of inclusion body hepatitis and hepatitis-hydropericardium syndrome in poultry: a comprehensive review. Front Vet Sci 9:963199 36304412 10.3389/fvets.2022.963199 PMC9592805

[3] Shah MS, Ashraf A, Khan MI, Rahman M, Habib M, Chughtai MI, Qureshi JA (2017) Fowl adenovirus: history, emergence, biology and development of a vaccine against hydropericardium syndrome. Arch Virol 162:1833–1843 28283816 10.1007/s00705-017-3313-5

[4] Schachner A, Matos M, Grafl B, Hess M (2018) Fowl adenovirus-induced diseases and strategies for their control - a review on the current global situation. Avian Pathol 47:111–126 28950714 10.1080/03079457.2017.1385724

[5] Niczyporuk JS (2018) Deep analysis of loop L1 HVRs1-4 region of the hexon gene of adenovirus field strains isolated in Poland. PLoS One 13:e0207668 30481218 10.1371/journal.pone.0207668 PMC6258537

[6] Schachner A, Gonzalez G, Endler L, Ito K, Hess M (2019) Fowl adenovirus (FAdV) recombination with intertypic crossovers in genomes of FAdV-D and FAdV-E, displaying hybrid serological phenotypes. Viruses 11:1094 31779121 10.3390/v11121094 PMC6950264

[7] Benkő M, Aoki K, Arnberg N, Davison AJ, Echavarría M, Hess M, Jones MS, Kaján GL, Kajon AE, Mittal SK, Podgorski II, San Martín C, Wadell G, Watanabe H, Harrach B, Consortium IR (2022) ICTV virus taxonomy profile: Adenoviridae 2022. J Gen Virol 103:001721 35262477 10.1099/jgv.0.001721 PMC9176265

[8] Niczyporuk JS (2016) Phylogenetic and geographic analysis of fowl adenovirus field strains isolated from poultry in Poland. Arch Virol 161:33–42 26446890 10.1007/s00705-015-2635-4

[9] Hosseini H, Najafi H, Fallah Mehrabadi MH, Gholamian B, Noroozi S, Ahmadi M, Ziafati Kafi Z, Sadri N, Hojabr Rajeoni A, Ghalyanchilangeroudi A (2021) Molecular detection of fowl adenovirus 7 from slaughtered broiler chickens in Iran: the first report. Iran J Vet Res 22:244–247 34777527 10.22099/ijvr.2021.37426.5452 PMC8573397

[10] Oliver-Ferrando S, Dolz R, Calderón C, Valle R, Rivas R, Pérez M, Biarnés M, Blanco A, Bertran K, Ramis A, Busquets N, Majó N (2017) Epidemiological and pathological investigation of fowl aviadenovirus serotypes 8b and 11 isolated from chickens with inclusion body hepatitis in Spain (2011–2013). Avian Pathol 46:157–165 27928940 10.1080/03079457.2016.1232477

[11] Mase M, Iseki H, Watanabe S (2021) Complete genome sequence of a fowl adenovirus D strain isolated from chickens with inclusion body hepatitis in Japan. Microbiol Resour Announc 10:e0094021 34792382 10.1128/MRA.00940-21 PMC8601138

[12] Yamaguchi M, Miyaoka Y, Hasan MA, Kabir MH, Shoham D, Murakami H, Takehara K (2022) Isolation and molecular characterization of fowl adenovirus and avian reovirus from breeder chickens in Japan in 2019–2021. J Vet Med Sci 84:238–243 34980758 10.1292/jvms.21-0616 PMC8920717

[13] Park DH, Lee HC, Youn HN, Ju HS, Kim KJ, Go SH, Lee DY, Lee JB, Lee SW, Song CS (2021) Genetic characterization and pathogenicity analysis of recently isolated fowl adenovirus 8b in Korea. Avian Dis 65:122–131 34339131 10.1637/aviandiseases-D-20-00097

[14] Zhuang Q, Wang S, Zhang F, Zhao C, Chen Q, Zhao R, Guo P, Ju L, Li J, Hou G, Chen X, Sun F, Wang K (2023) Molecular epidemiology analysis of fowl adenovirus detected from apparently healthy birds in eastern China. BMC Vet Res 19:5 36624468 10.1186/s12917-022-03545-5 PMC9827690

[15] Mete A, Armien AG, Rejmanek D, Mott M, Crossley BM (2021) Emergence of fowl aviadenovirus C-4 in a backyard chicken flock in California. J Vet Diagn Invest 33:806–809 34085872 10.1177/10406387211019962 PMC8229832

[16] De la Torre D, Nuñez LFN, Santander Parra SH, Astolfi-Ferreira CS, Piantino Ferreira AJ (2018) Molecular characterization of fowl adenovirus group I in commercial broiler chickens in Brazil. Virusdisease 29:83–88 29607363 10.1007/s13337-018-0430-z PMC5877850

[17] Wang T, Meng F, Chen C, Shen Y, Li P, Xu J, Feng Z, Qu X, Wang F, Li B, Liu M (2023) Pathogenicity and epidemiological survey of fowl adenovirus in Shandong Province from 2021 to 2022. Front Microbiol 14:1166078 37234528 10.3389/fmicb.2023.1166078 PMC10206033

[18] Mohamed MHA, El-Sabagh IM, Abdelaziz AM, Al-Ali AM, Alramadan M, Lebdah MA, Ibrahim AM, Al-Ankari AS (2018) Molecular characterization of fowl aviadenoviruses species D and E associated with inclusion body hepatitis in chickens and falcons indicates possible cross-species transmission. Avian Pathol 47:384–390 29587493 10.1080/03079457.2018.1457769

[19] Kim HR, Song HS, Jang I, Thai TN, Kim HS, Kwon YK, Her M (2025) Nationwide surveillance of fowl adenovirus infection and coinfection with other diseases on slaughter broiler in South Korea. Transbound Emerg Dis 2025:9353432 40302733 10.1155/tbed/9353432 PMC12016797

[20] Xu AH, Sun L, Tu KH, Teng QY, Xue J, Zhang GZ (2021) Experimental co-infection of variant infectious bursal disease virus and fowl adenovirus serotype 4 increases mortality and reduces immune response in chickens. Vet Res 52:61 33926543 10.1186/s13567-021-00932-y PMC8082832

[21] Brown Jordan A, Blake L, Bisnath J, Ramgattie C, Carrington CV, Oura CAL (2019) Identification of four serotypes of fowl Adenovirus in clinically affected commercial poultry co-infected with Chicken Infectious Anaemia Virus in Trinidad and Tobago. Transbound Emerg Dis 66:1341–1348 30817083 10.1111/tbed.13162

[22] Liu L, Gao W, Chang J, Liu J, Huang Z, Sun W, Song Y, Li X (2025) Co-infection of Chicken Infectious Anemia Virus and fowl Adenovirus serotype E8b increases mortality in chickens. Viruses 17:620 40431632 10.3390/v17050620 PMC12115589

[23] Zhang F, Li P, Shi H, Zhu H, Wang X, Liu X, Wang T, Song Q, Li Z, Liu C (2026) Epidemiological characterization of fowl Adenovirus in China from 2021 to June 2025. Poult Sci 105:106696 41775158 10.1016/j.psj.2026.106696 PMC12970399

[24] Kim MS, Lim TH, Lee DH, Youn HN, Yuk SS, Kim BY, Choi SW, Jung CH, Han JH, Song CS (2014) An inactivated oil-emulsion fowl Adenovirus serotype 4 vaccine provides broad cross-protection against various serotypes of fowl Adenovirus. Vaccine 32:3564–3568 24662704 10.1016/j.vaccine.2014.03.015

[25] Meulemans G, Boschmans M, Berg TP, Decaesstecker M (2001) Polymerase chain reaction combined with restriction enzyme analysis for detection and differentiation of fowl adenoviruses. Avian Pathol 30:655–660 19184959 10.1080/03079450120092143

[26] Liu M, Chen YY, Twu NC, Wu MC, Fang ZS, Dubruel A, Chang SC, Wu CF, Lo DY, Chen HW (2024) A novel goose-origin Tembusu virus exhibits pathogenicity in day-old chicks with evidence of direct contact transmission. Poult Sci 103:103332 38128459 10.1016/j.psj.2023.103332 PMC10776645

[27] Reed LJ, Muench H (1938) A simple method of estimating fifty per cent endpoints. Am J Epidemiol 27:493–497

[28] Tamura K, Stecher G, Kumar S (2021) MEGA11: molecular evolutionary genetics analysis version 11. Mol Biol Evol 38:3022–3027 33892491 10.1093/molbev/msab120 PMC8233496

[29] Lole KS, Bollinger RC, Paranjape RS, Gadkari D, Kulkarni SS, Novak NG, Ingersoll R, Sheppard HW, Ray SC (1999) Full-length Human Immunodeficiency Virus type 1 genomes from subtype C-infected seroconverters in India, with evidence of intersubtype recombination. J Virol 73:152–160 9847317 10.1128/jvi.73.1.152-160.1999 PMC103818

[30] Archetti I, Horsfall FL Jr. (1950) Persistent antigenic variation of Influenza A viruses after incomplete neutralization in ovo with heterologous immune serum. J Exp Med 92:441–462 10.1084/jem.92.5.441 PMC2135986 14778924

[31] Li YT, Chen TC, Lin SY, Mase M, Murakami S, Horimoto T, Chen HW (2020) Emerging lethal infectious bronchitis coronavirus variants with multiorgan tropism. Transbound Emerg Dis 67:884–893 31682070 10.1111/tbed.13412 PMC7138078

[32] Günes A, Marek A, Grafl B, Berger E, Hess M (2012) Real-time PCR assay for universal detection and quantitation of all five species of fowl adenoviruses (FAdV-A to FAdV-E). J Virol Methods 183:147–153 22561984 10.1016/j.jviromet.2012.04.005

[33] Chang HM (2004) DNA grouping, serotyping, pathogenicity, and serological survey of the fowl adenovirus isolated in Taiwan. Master thesis, National Taiwan University, Graduate Institute of Veterinary Medicine, School of Veterinary Medicine

[34] Mo J (2021) Historical investigation of fowl adenovirus outbreaks in South Korea from 2007 to 2021: a comprehensive review. Viruses 13:2256 34835062 10.3390/v13112256 PMC8621494

[35] Chen L, Yin L, Peng P, Zhou Q, Du Y, Zhang Y, Xue C, Cao Y (2020) Isolation and characterization of a novel fowl adenovirus serotype 8a strain from China. Virol Sin 35:517–527 31792739 10.1007/s12250-019-00172-7 PMC7736427

[36] Lv L, Lu H, Wang K, Shao H, Mei N, Ye JQ, Chen HJ (2021) Emerging of a novel natural recombinant fowl adenovirus in China. Transbound Emerg Dis 68:283–288 32657542 10.1111/tbed.13730

[37] Matos M, Grafl B, Liebhart D, Hess M (2016) The outcome of experimentally induced inclusion body hepatitis (IBH) by fowl aviadenoviruses (FAdVs) is crucially influenced by the genetic background of the host. Vet Res 47:69 27356980 10.1186/s13567-016-0350-0 PMC4928300

[38] Liu A, Zhang Y, Wang J, Cui H, Qi X, Liu C, Zhang Y, Li K, Gao L, Wang X, Gao Y, Pan Q (2022) Complete genome analysis and animal model development of fowl adenovirus 8b. Viruses 14:1826 36016448 10.3390/v14081826 PMC9416599

